# Supplementary material for: Clinical characteristics and prognostic nomogram analysis of patients with dual primary cancers with first gastric cancer: a retrospective study in China
Source: PeerJ. 2023 May 1;11:e15278. doi: 10.7717/peerj.15278 (PMC10158755; doi:10.7717/peerj.15278)
Supplement: Supplemental Information 2 [file peerj-11-15278-s002.docx]

According to the sequence of result arrangement, we list the involved statistical methods and specific points in turn.

**Result1. General Features of DPCFGC Patients**

① Method of statistics: incidence, composition ratio and median

② The corresponding test statistic: ratio of synchronous and metachronous patients; ratio of male and female patients; percentage of patients in different diagnostic intervals; percentage of patients in different systems and organs; percentage of patients in different lifestyles, family history of cancers, history of chronic diseases

③ Sample size: A total of 78 cases

**Result2. Pathological Characteristics of GC and SPCs**

① Method of statistics: composition ratio, χ2 test or Fisher exact probability test

② The corresponding test statistic: Pathological type of GC and SPCs; Histological grade of GC and SPCs; Pathological stage of GC and SPCs

③ Sample size: A total of 78 cases (37 cases of synchronous and 41 cases of metachronous patients)

④ The exact p-value: Pathological type of GC, Histological grade of GC, Pathological stage of GC, Pathological type of SPCs, Histological grade of SPCs and Pathological stage of SPCs had the exact *P*-values of 0.084, 0.683, < 0.001, 0.588, 0.995 and 0.003, respectively.

⑥ Application condition: When T _min_ ≥ 5 and N ≥ 40, the common χ2 test was adopted; When 1 ≤ T _min_ ≤ 5 and N ≥ 40, the corrected χ2 test was adopted; When T _min_ < l; 1, N < 40. Fisher's exact probability method was adopted.

**Result3. Treatment Modalities of GC and SPCs**

① Method of statistics: composition ratio

② The corresponding test statistic: The proportion of different treatment modalities of GC and SPC

③ Sample size: A total of 78 cases (37 cases of synchronous and 41 cases of metachronous patients)

**Result4. Analysis of Survival Status and Prognostic Factors**

① Method of statistics: Univariate and multivariate COX analysis

② The corresponding test statistic: See Table 1, 2

③ Sample size: A total of 78 cases

④ The exact *P*-value, degrees of freedom and 95.0% CI: See Table 1, 2

Table 1 The results of multivariate COX analysis

|  | B | SE | Wolf | Degrees of freedom | *P*-value | HR | 95.0% CI | |
| --- | --- | --- | --- | --- | --- | --- | --- | --- |
|  |  |  |  |  |  |  | lower limit | upper limit |
| Gender (female vs. male) | -0.259 | 0.254 | 1.044 | 1 | 0.307 | 0.772 | 0.469 | 1.269 |
| BMI (＞25 vs. ≤25) | -0.162 | 0.465 | 0.122 | 1 | 0.727 | 0.850 | 0.342 | 2.115 |
| Smoking (yes vs. no) | 0.595 | 0.279 | 4.548 | 1 | 0.033^*^ | 1.814 | 1.049 | 3.135 |
| Alcoholism (yes vs. no) | 0.633 | 0.293 | 4.665 | 1 | 0.031^*^ | 1.883 | 1.060 | 3.343 |
| Family history of cancers  (yes vs. no) | -0.010 | 0.466 | 0.000 | 1 | 0.983 | 0.990 | 0.397 | 2.468 |
| History of hypertension  (yes vs. no) | 0.616 | 0.325 | 3.599 | 1 | 0.058 | 1.851 | 0.980 | 3.497 |
| History of coronary heart disease (yes vs. no) | 1.155 | 0.417 | 7.687 | 1 | 0.006^*^ | 3.174 | 1.403 | 7.180 |
| History of diabetes (yes vs. no) | 0.646 | 0.523 | 1.524 | 1 | 0.217 | 1.908 | 0.684 | 5.319 |
| History of COPD (yes vs. no) | 1.376 | 0.621 | 4.904 | 1 | 0.027^*^ | 3.960 | 1.171 | 13.384 |
| Diagnostic age of GC (＞60 vs. ≤60) | 0.546 | 0.246 | 4.919 | 1 | 0.027^*^ | 1.726 | 1.066 | 2.796 |
| Histological grade of GC  (high vs. low+ middle) | -0.405 | 0.592 | 0.468 | 1 | 0.494 | 0.667 | 0.209 | 2.128 |
| Pathological stage of GC  (III vs. I+ II) | 0.736 | 0.252 | 8.525 | 1 | 0.004^*^ | 2.089 | 1.274 | 3.424 |
| Whether to operate for GC  (yes vs. no) | -2.318 | 0.368 | 39.783 | 1 | <0.001^*^ | 0.098 | 0.048 | 0.202 |
| Whether adjuvant therapy for GC (yes vs. no) | -0.078 | 0.257 | 0.091 | 1 | 0.763 | 0.925 | 0.559 | 1.532 |
| Diagnostic interval (＞6 vs. ≤6) | -1.202 | 0.249 | 23.293 | 1 | <0.001^*^ | 0.301 | 0.184 | 0.490 |
| Diagnostic age of SPCs(＞65 vs. ≤65) | 0.346 | 0.238 | 2.123 | 1 | 0.145 | 1.414 | 0.887 | 2.252 |
| Histological grade of SPCs (high vs. low+ middle) | 0.415 | 0.270 | 2.356 | 1 | 0.125 | 1.514 | 0.892 | 2.570 |
| Pathological stage of SPCs  (III+ IV vs. I+ II) | 0.792 | 0.244 | 10.542 | 1 | 0.001^*^ | 2.209 | 1.369 | 3.564 |
| Whether to operate for SPCs (yes vs. no) | -0.503 | 0.245 | 4.218 | 1 | 0.040^*^ | 0.605 | 0.374 | 0.977 |
| Whether adjuvant therapy for SPCs (yes vs. no) | -0.230 | 0.239 | 0.925 | 1 | 0.336 | 0.795 | 0.497 | 1.270 |
| Note: ^*^*P* <0.05 | | | | | | | | |

Table 2 The results of Univariate COX analysis

|  | B | SE | Wolf | Degrees of freedom | *P*-value | HR | 95.0% CI | |
| --- | --- | --- | --- | --- | --- | --- | --- | --- |
|  |  |  |  |  |  |  | lower limit | upper limit |
| Smoking (yes vs. no) | 0.749 | 0.407 | 3.392 | 1 | 0.066 | 2.115 | 0.953 | 4.695 |
| Alcoholism (yes vs. no) | 0.788 | 0.417 | 3.575 | 1 | 0.059 | 2.198 | 0.972 | 4.972 |
| History of coronary heart disease (yes vs. no) | 0.052 | 0.582 | 0.008 | 1 | 0.928 | 1.054 | 0.337 | 3.296 |
| History of COPD (yes vs. no) | 0.055 | 0.847 | 0.004 | 1 | 0.948 | 1.056 | 0.201 | 5.552 |
| Diagnostic age of GC  (＞60 vs. ≤60) | 0.557 | 0.287 | 3.775 | 1 | 0.052 | 1.746 | 0.995 | 3.064 |
| Pathological stage of GC  (III vs. I+ II) | 0.724 | 0.303 | 5.703 | 1 | 0.017^*^ | 2.063 | 1.139 | 3.736 |
| Whether to operate for GC  (yes vs. no) | -1.261 | 0.494 | 6.510 | 1 | 0.011^*^ | 0.283 | 0.108 | 0.747 |
| Diagnostic interval  (＞6 vs. ≤6) | -1.025 | 0.373 | 7.566 | 1 | 0.006^*^ | 0.359 | 0.173 | 0.745 |
| Pathological stage of SPCs  (III+ IV vs. I+ II) | 0.617 | 0.291 | 4.493 | 1 | 0.034^*^ | 1.853 | 1.048 | 3.279 |
| Whether to operate for SPCs  (yes vs. no) | -0.371 | 0.310 | 1.431 | 1 | 0.232 | 0.690 | 0.376 | 1.267 |
| Note: ^*^ *P* <0.05 | | | | | | | | |

**Result5. Construction and Validation of Prognostic Nomogram**

① Method of statistics: receiver operating characteristic curves (ROC)

② The corresponding test statistic: 1-, 2-, 3-year overall

③ Sample size: A total of 78 cases

④ R software packages involved: prognostic model constructed by survival and survminer packages; nomogram drew by rms package; ROC curves drew by timeROC package, calibration curves drew by rms package; decision curve analysis drew by stdCa package.

**Result6. Association of Prognostic Factors and Model with OS**

① Method of statistics: Kaplan-Meier analysis and log-rank test

② The corresponding test statistic: Pathological stage of GC, whether to operate for GC, diagnostic interval, pathological stage of SPCs and total risk

③ Sample size: A total of 78 cases

④ The exact *P*-value, HR, 95.0% CI: See Table 3

Table 3 The results of Kaplan-Meier analysis

|  | HR | *P*-value | 95.0% CI | |
| --- | --- | --- | --- | --- |
|  |  |  | lower limit | upper limit |
| Pathological stage of GC  (III vs. I+ II) | 2.114 | 0.002^*^ | 1.290 | 3.463 |
| Whether to operate for GC  (yes vs. no) | 0.092 | < 0.001^*^ | 0.045 | 0.188 |
| Diagnostic interval  (＞6 vs. ≤6) | 0.295 | < 0.001^*^ | 0.182 | 0.480 |
| Pathological stage of SPCs  (III+ IV vs. I+ II) | 2.245 | < 0.001^*^ | 1.393 | 3.618 |
| Total risk  (High risk vs. low risk) | 5.721 | < 0.001^*^ | 3.353 | 9.761 |
| Note: ^*^ *P* <0.05 | | | | |
